# Contrasting resting-state fMRI abnormalities from sickle and non-sickle anemia

**DOI:** 10.1371/journal.pone.0184860

**Published:** 2017-10-05

**Authors:** Julie Coloigner, Yeun Kim, Adam Bush, Soyoung Choi, Melissa C. Balderrama, Thomas D. Coates, Sharon H. O’Neil, Natasha Lepore, John C. Wood

**Affiliations:** 1 CIBORG laboratory, Division of Radiology, Children’s Hospital, Los Angeles, California, United States of America; 2 Department of Biomedical Engineering, University of Southern California, Los Angeles, California, United States of America; 3 Neuroscience Graduate Program, University of Southern California, Los Angeles, California, United States of America; 4 Division of Hematology, Oncology and Blood and Marrow Transplantation, Children’s Hospital, Los Angeles, California, United States of America; 5 Department of Pediatrics, Keck School of Medicine, University of Southern California, Los Angeles, California, United States of America; 6 Division of Neurology, Children’s Hospital, Los Angeles, California, United States of America; 7 The Saban Research Institute, Children’s Hospital, Los Angeles, California, United States of America; 8 Division of Cardiology, Children’s Hospital, Los Angeles, California, United States of America; University of Toronto, CANADA

## Abstract

Sickle cell disease (SCD) is a chronic blood disorder that is often associated with acute and chronic cerebrovascular complications, including strokes and impaired cognition. Using functional resting state magnetic resonance images, we performed whole-brain analysis of the amplitude of low frequency fluctuations (ALFF), to detect areas of spontaneous blood oxygenation level dependent signal across brain regions. We compared the ALFF of 20 SCD patients to that observed in 19 healthy, age and ethnicity-matched, control subjects. Significant differences were found in several brain regions, including the insula, precuneus, anterior cingulate cortex and medial superior frontal gyrus. To identify the ALFF differences resulting from anemia alone, we also compared the ALFF of SCD patients to that observed in 12 patients having comparable hemoglobin levels but lacking sickle hemoglobin. Increased ALFF in the orbitofrontal cortex and the anterior and posterior cingulate cortex and decreased ALFF in the frontal pole, cerebellum and medial superior frontal gyrus persisted after accounting for the effect of anemia. The presence of white matter hyperintensities was associated with depressed frontal and medial superior frontal gyri activity in the SCD subjects. Decreased ALFF in the frontal lobe was correlated with decreased verbal fluency and cognitive flexibility. These findings may lead to a better understanding of the pathophysiology of SCD.

## Introduction

Sickle cell disease (SCD) is a chronic blood disorder caused by a mutation in the beta hemoglobin gene [1] that affects millions of people around the world. In the U.S., the disease affects approximately 90,000 people, and 1,000 babies are born with SCD every year [2]. With this disease, hemoglobin polymerizes upon deoxygenation, causing red blood cells to form a “sickle” shape and become rigid and fragile [3]. Sickle red blood cells cause vaso-occlusion and hemolysis which produce free hemoglobin and result in vascular damage via mechanical and oxidative stress. Stroke, transient ischemic attack and brain atrophy are common in SCD, which lead high rates of significant mortality and morbidity. Previous magnetic resonance imaging (MRI) studies have reported delay in brain gray matter development in children with SCD [4, 5], as well as regional cortical thinningin the precuneus and posterior cingulate, which are possible manifestations of chronic insufficient oxygen delivery to watershed tissue [6]. Chronic vascular insufficiency also causes diffuse white matter damage [7]. White matter hyperintensities (WMH) or silent stroke, detected by T2-weighted images, are commonly observed in SCD patients [8–11]. Additionally, lower white matter volume has been detected, especially in watershed regions, in patients with SCD [12]. Despite routine neurovascular screening and aggressive use of hydroxyurea prophylaxis in these patients (https://www.nhlbi.nih.gov/health-pro/guidelines/sickle-cell-disease-guidelines/), the prevalence of silent stroke increases linearly with age, reaching 50% by the age of 30 [13]. Several studies have linked silent stroke and white matter disease to cognitive deficits in children with SCD, including lower verbal intelligence quotients, poor math performance and visuo-motor impairment in children with SCD [14–16]. Therefore, there is a clear need to investigate novel neuroimaging markers of the disease in SCD patients.

Functional Blood oxygenation level dependent (BOLD) MRI is one of the most widely utilized non-invasive imaging techniques to indirectly measure brain function. The BOLD signal is sensitive to changes in the presence of deoxygenated blood, reflecting the balance between oxygen consumption and oxygen supply. Neural activity results in oxygen utilization which transiently decreases BOLD signal. This is followed by neurovascular coupling, triggering an increase in blood flow, which increases BOLD signal, and thereby creating signal fluctuations. Synchronous fluctuations of BOLD signal at rest have been shown in different cerebral regions, constituting well reproducible resting-state functional networks (RSNs) [17, 18]. More than ten networks have been consistently identified across subjects [19, 20]. Among them, the default mode network, which is the principle resting network prominent in all subjects, is disturbed in many disease state and comprises mainly the posterior cingulate cortex (PCC), precuneus, medial prefrontal cortex (mPFC), and the angular/lateral parietal cortex [21].

The default mode network has been assessed using the amplitude of low-frequency fluctuations (ALFF) method [22], which represents the the regional intensity of spontaneous fluctuations, and is calculated by the square root of the power spectrum in a low-frequency range [23]. Higher ALFF in resting-state have been shown in regions of the default mode network [24]. Instead of identifying specific abnormal networks by calculating the connectivity between specific regions, the ALFF allows whole brain localization of functional alterations. This method has been used to study various diseases including Alzheimer’s disease [25], depression [26], drug addiction [27], subcortical ischemic vascular dementia [28] and ischemic penumbra [29]. These studies demonstrated the feasibility of using fMRI analysis as a neurological index to show group differences between healthy controls and patients with neurological diseases. Moreover, ALFF of specific functional areas can be correlated with behavioral performance [30–32] to understand the relationship between neuronal activation and neurocognitive functioning.

In this study, we hypothesized that patients with SCD would differ from controls in different regional brain activity because of their chronic anemia, microvascular damage and known neurocognitive deficits [8]. As the effects of chronic blood disorders on the brain may be global, a whole-brain ALFF analysis was performed. We have previously demonstrated that chronic anemia produces compensatory increases in cerebral blood flow such that resting oxygen delivery to the brain is preserved [33, 34]. However, resting cerebral hyperemia leaves the brain with low cerebral vascular reserve [35, 36]. Acute interruptions in oxygen delivery (acute hemoglobin drop, hypoxia) or increased metabolic demand (fever, seizure) can provoke microvascular damage and stroke in patients with low cerebral vascular reserve. To help discern the impact of anemia, alone, from the impact of anemia plus abnormal red blood cell properties, we also included anemic control subjects (ACTL) who had normal red blood cells but comparable hemoglobin levels as SCD patients. Patients with non-sickle anemia are at risk for silent strokes [37, 38] but it is not known whether their stroke risk and neurocognitive deficits are as severe as in SCD patients. We postulated that resting state BOLD activity in our SCD and ACTL cohorts would both differ from control subjects, but that SCD patients would have unique features resulting from more severe microvascular damage. We also investigated the relationship between ALFF changes and neurocognitive scores to potentially discern physiologic from pathophysiologic changes in brain activation.

## Materials and methods

### Participants

Patients with SCD were enrolled from Children’s Hospital Los Angeles (CHLA) between January 2012 and September 2015. The Committee on Clinical Investigation at Children’s Hospital Los Angeles approved the protocol (CCI#11–00083). All subjects older than 18 provided signed, informed consent. For children between 13 and 18 years of age, consent was obtained from the research subject and one of their parents. Written assent, plus parental consent, was obtained from all children younger than 13 years of age. We restricted the population to adolescents and young adults without previous overt strokes or known cerebrovascular disease. Other exclusion criteria were the following: 1) pregnancy; 2) occurrence of acute chest or pain crisis hospitalization within the past month; and 3) additional conditions such as epilepsy or traumatic brain injury. Thus, all patients were in their steady-state. Study subjects were not evaluated by a neurologist, but no focal neurologic deficits were documented in their medical records. The population sample consisted of 20 SCD patients, 12 ACTL patients and 19 healthy control (CTL) subjects. The control group consisted of five white Hispanic participants, one white Middle Eastern participant and 13 African American non-Hispanic participants. The ACTL group consisted of seven patients with β-thalassemia major, two patients with chronically transfused Eβ thalassemia, one patient with thalassemia intermedia, one patient with autoimmune hemolytic anemia and one patient with dyserythropoetic anemia. Eleven of the ACTL patients were chronically transfused, for a duration of 15.6 +/- 5.8 years [5.9–24.4 years]. Indications for chronic transfusion in the ACTL group were severe anemia and adverse effects of ineffective erythropoiesis. Ethnically, the ACTL cohort was composed of four Asian patients, four white Hispanic patients, three Indian or Pakistan patients and one Mediterranean patients. The sickle cell disease patient genotypes were SS (17 patients), Sβ_0_ phenotype (two patients), and SC (one patient). SCD subjects were African American (14 patients), white Hispanic (five patients) or Middle Eastern (one patient). Twelve of the SCD patients were receiving regular blood transfusions and eight were not routinely transfused; indications for blood transfusion were history of abnormal transcranial Doppler examination (eight patients), acute chest syndrome (two patients), severe anemia secondary to renal disease (one patient) and unknown reasons (one patient). SCD patients had been transfused for 7.6 +/- 3.0 years (range 3.5–13.6 year). All patients receiving chronic transfusions (ACTL and SCD) were maintained on iron chelation with deferasirox. Seven of the eight nontransfused SCD patients were prescribed hydroxyurea. Four of the seven patients had increased mean corpuscular volume suggesting good hydroxyurea response. Hemoglobin F was increased in the nontransfused SCD population (because of hydroxyurea usage) but not significantly increased in any of the chronically transfused patients (Table 1). The three groups were matched in terms of sex and age. However, the ACTL group had lower maternal education than the CTL group. All chronically transfused patients were studied within 6 hours prior to their regularly scheduled blood transfusion when their hemoglobin levels were most comparable to the nontransfused patients. Hemoglobin levels were 10.6±1.4g/dL in the nontransfused SCD patients and 9.0±1.0g/dL in the transfused SCD patients. The mean hemoglobin S percentage was 70.0% in the nontransfused SCD group whereas this quantity was reduced to 20.1% in the transfused SCD group. Transfused SCD patients had the largest reticulocyte count and LDH values, but cell free hemoglobin was actually highest in the ACTL group. Demographic and clinical variables among CTL, ACTL and SCD patients as well as statistical comparisons between the groups are given in Table 1.

**Table 1:** Demographic and clinical variables among healthy controls (CTL), anemic control subjects (ACTL) and patients with sickle cell disease (SCD) including the transfused (NTx) and the non-transfused patients.

	CTL	ACTL	SCD	SCD (NTx)	SCD (Tx)
**# Subjects**	19	12	20	8	12
**Age**^†^	22.05 (17–41.8)	20.8 (12.6–31.4)	19.7 (12.4–34.4)	22 (16.7–44.4)	16.8 (12.4–25.4)
	13 African	4 East Asian	14 African		
**Racial/Ethnic**	5 Hispanic	4 Hispanic	5 Hispanic		
**Background**	1 Middle Eastern	3 India Pakistan	1 Middle Eastern		
		1 Mediterranean			
**Sex**	13F, 6M	7F, 5M	11F, 9M	4F, 4M	7F, 5M
**Mother education**	3.4 ± 1.3	2.2 ± 1.4	2.7 ± 1.3	3.0±0.8	2.5±1.3
**SBP (mm Hg)**	116 ± 10	111 ± 9	110 ± 10	110 ± 6.7	110 ± 11.7
**DBP (mm Hg)**	66 ± 9	62 ± 9	60 ± 7	60 ± 5.1	60 ± 8
**MAP (mmHg)**	83 ± 10	78 ± 8	76 ± 8	76 ± 8	77 ± 7
**Pulse (bpm)**	80 ± 15	80 ± 12	84 ± 11	74 ± 10	89 ± 8
**Ht (cm)**	166 ± 8	163 ± 10	161 ± 8	163 ± 7	161 ± 9
**Wt (kg)**	66 ± 14	60 ± 11	61 ± 15	67 ± 14	57 ± 15
**BMI (kg/m**^**2**^**)**	23.8 ± 5.1	22.4 ± 2.6	23.2 ± 5.5	25.4 ± 5.6	21.9 ± 5.1
**O**_**2**_ **Sat (%)**	99.4 ± 0.8	99.1 ± 1.1	97.7 ± 2.0	96.9 ± 2.5	98.2 ± 1.5
**Hb (g/dl)**	13.8 ± 1.5	10.2 ± 1.0	9.7 ± 1.4	10.6 ± 1.4	9.0 ± 1.0
**WBC**	5.8 ± 1.6	6.4 ± 1.9	10.4 ± 5.5	6.7 ± 2.7	12.9 ± 5.3
**Plt**	253 ± 63	244 ± 107	250 ± 84	255 ± 64	247 ± 97
**MCV**	87.3 ± 5.0	83.1 ± 4.5	88.2 ± 11.3	90.7 ± 17.9	86.6 ± 3.1
**Retic (%)**	1.4 ± 0.7	1.4 ± 1.9	10.2 ± 6.5	9.7 ± 7.6	11.6 ± 5.9
**LDH (U/L)**	542 ± 88	450 ± 196	994 ± 546	751 ± 385	1156 ± 591
**Plasma Hb**	4.6 ± 2.6	22.9 ± 25.5	17.3 ± 15.7	13.0 ± 8.6	20.1 ± 18.9
**Hb S (%)**	11.2 ± 17.9	0	41.5 ± 27.4	70.0 ± 17.8	22.5 ± 10.6
**Hb F (%)**	0.9 ± 2.8	1.9 ± 2.5	5.9 ± 8.2	11.9 ± 10.4	2.0 ± 2.4
	14 AA	10 AA	17 SS	5 SS	12 SS
**Hb type**	5 AS	2 AE	2 Sβ_0_	2 Sβ_0_	
			1 SC	1 SC	
**Hydroxyurea**	0	0	7	7	0

SBP indicates the systolic blood pressure; DBP, diastolic blood pressure; MAP, mean arterial pressure; Pulse, heart rate; Ht, hematocrit; Wt, weight; BMI, body mass index; O_2_ sat; oxygen saturation; Hb, hemoglobin; WBC, white blood cell count; Plt, platelets; MCV, mean platelet volume; Retic, reticulocytes; LDH, lactose dehydrogenase; Hb S, concentration of Hemoglobin S; Hb F, concentration of Hemoglobin F.

^†^ expressed as median (range): the other variables are shown as mean ± standard deviation. The Chi-square test was used for gender. Other demographic and laboratory parameters were compared using Student’s t test. Bold text indicates a statistically significant difference. Maternal educational attainment was assessed by asking patients to choose their highest educational level completed from the following choices: 1 = less than high school; 2 = high school or GED completed; 3 = some college or technical school; 4 = college graduate and 5 = graduate school.

### MRI acquisition

All participants underwent a MR study using a 3T Philips Achieva with an 8-element phased-array headcoil at CHLA. The 3D T1-weighted images were acquired covering the whole brain (160 sagittal slices) with TR = 8.20 ms, TE = 3.8 ms, flip angle = 8°, in-plane resolution = 1 mm × 1 mm, FOV = 256 mm × 224 mm and thickness/gap = 1.0/0 mm. In order to identify WMHs, the 3D T2-weighted images were obtained with the parameters: TR = 4.8 s, TE = 255 ms, flip angle = 90°, in-plane resolution = 1 mm × 1 mm, FOV = 256 mm × 256 mm and thickness/gap = 1.3/0 mm. A cerebral MRA using three-dimensional time-of- flight angiography of the circle of Willis was acquired using TR = 23 ms, TE = 3.5 ms, 150 slices, 0.7 mm thick and a directional field of view of 10.5 cm. Phase contrast measurements of total cerebral blood flow were derived from a single axial phase contrast image positioned immediately superior to the carotid bifurcation and angled as perpendicular to the internal carotid arteries as possible. Imaging parameters include a repetition time 12.3 ms, echo time 7.5 ms, field of view 260x260 mm, slice thickness 5 mm, signal averages 10, acquisition matrix 204 x 201, reconstruction matrix 448 x 448, bandwidth 244 Hz/pixel, and velocity encoding gradient of 200 cm/s [34].

During resting-state fMRI scanning, subjects were instructed to close their eyes, remain as still as possible,to not think of anything systematically, and to not fall asleep. The functional images were acquired with the following parameters: TR = 2000 ms, TE = 50 ms, flip angle = 90°, in-plane resolution = 2.3 mm × 2.3 mm, FOV = 220 mm × 220 mm, 26 axial slices, thickness/gap = 5/0 mm. A total of 240 volumes were collected in 8 minutes.

### Analysis of anatomic and phase contrast images

Angiography, 3D T1, and 3D T2 images were analyzed by a single, board-certified radiologist. The WMH were classified as ≥ 3 mm lesions on T2 FLAIR observed in two orthogonal planes, with or without corresponding changes on T1. Since patients with known strokes were excluded, WMH were considered “silent” irrespective of neurocognitive testing results. Since isolated WMH are relatively common in children [39] and increase in frequency with age [40, 41], more than one lesion per decade of age was required to be considered pathological.

Phase contrast images were analyzed using custom MATLAB routines as described previously [33, 34]. Briefly, an operator placed a single point within each of the four major cerebral vessels. Boundaries were automatically segmented from the complex difference images using a Canny edge detector, grown by a single voxel, and thresholded at values three times greater than the noise floor. All contours were confirmed by the operator and manually traced in the event of automatic algorithm failure (<5%).

### Neuropsychological assessment

All participants completed a three- to four-hour battery of standardized neuropsychological tests assessing intelligence, working memory, processing speed, executive function, verbal and nonverbal memory, and social-emotional functioning. Testing was performed by a neuropsychologist and by doctoral trainees under the neuropsychologist’s supervision. Intelligence was measured with the Wechsler Abbreviated Scale of Intelligence, Second Edition (WASI-II) [42]. Working memory and processing speed were measured with the Digit Span, Coding and Symbol Search subtests of the Wechsler Intelligence Scale for Children, Fourth Edition (WISC-IV) [43] or the Wechsler Adult Intelligence Scale, Fourth Edition (WAIS-IV) [44]. Executive functions were measured with the Trail Making Test, Color-Word Interference and Verbal Fluency subtests of the Delis-Kaplan Executive Function System (D-KEFS) [45]. Verbal Memory was measured with the age appropriate version of the California Verbal Learning Test (CVLT) [46]. One ACTL patient, 2 CTL subjects and 6 patients with SCD completed CVLT- C. The CVLT-II was used for participants who were older than 16 years old. Nonverbal memory was measured with the Rey Osterrieth Complex Figure Test (Rey-O) [47]. Social emotional functioning was assessed with either the self- or parent-report version of the Behavior Assessment System for Children, Second Edition (BASC 2) [48]. Forty-three out of the 51 subjects completed all neurocognitive assessments. Five SCD patients and three CTL subjects did not complete Symbol Search processing speed test, because it was added to the battery after the start of the study.

### Image processing

Quantitative volumetric analysis was performed on the 3D T1 images, using BrainSuite in a semi-automated fashion to classify tissue types, extract and render the surfaces of the inner and pial cortices, as described in previous study [12]. Cerebral volumes and average cortical thickness were calculated for the whole brain and each lobe.

The resting-state fMRI data were preprocessed with the FMRIB Software Library (FSL) [49–51], using standard spatial functional pipeline. The first two volumes from each subject were discarded to allow the signal to reach equilibrium and the participants to adapt to the scanning noise. The 238 remaining volumes were corrected for the acquisition time delay between slices. Rigid realignment with FSL’s MCFLIRT was then performed to register the volumes to the mean fMRI volume to compensate for subject motion and ensure voxel-to-voxel correspondence across time. The head motion RMS was calculated by applying the affine matrices provided by the previous algorithm to a subset of voxels [52]. Only participants with a head motion root-mean-square error less than 1.5mm were included in this analysis. The mean fMRI volume was co-registered to the participant’s structural image, using a rigid body transformation model (6 parameters). The structural image was then transformed to the MNI template space, using the linear and non-linear FSL registration algorithms, FLIRT and FNIRT. For each patient, three image transformations were computed: the transformation from each volume to the mean fMRI volume, the transformation from the mean functional volume to the structural volume, and the transformation from the individual structural volume to the standard MNI structural template. By concatenating the above three transformations sequentially, we obtained a direct transformation from each initial functional volume to the standard MNI space. Finally, the registered fMRI volumes were normalized and smoothed using an 8mm × 8 mm × 8mm Gaussian kernel.

A general linear model analysis was then used to remove residual noise and motion in each voxel as a function of 17 regressors. The models evaluated included the standard 6 motion parameters (3 translations +3 rotations) estimated by FSL’s MCFLIRT as well as their temporal derivatives (6 parameters), computed by backwards differences. In addition, 5 principal components from noisy regions-of-non-interest, namely white matter, cerebral spinal fluid, and matter outside the brain, were used as regressors, which were calculated by the CompCor method [53] as implemented in Nilearn [54].

### ALFF analysis

The ALFF maps [55] were calculated by band-pass filtering the time-series of the functional volumes in a window of 0.008–0.09 Hz, estimating the power spectrum via a fast Fourier transform, and taking the averaged square root of this spectrum. To reduce global effects of variability across the participants, the ALFF at each voxel location was normalized by the mean whole-brain ALFF for that subject. All these steps were performed with FSL functions.

### Statistical analysis

#### Within-group ALFF analysis

For within-group whole-brain ALFF patterns, one-sample *t*-tests were performed on the individual ALFF maps in a voxelwise manner for the SCD, ACTL and CTL groups. Thresholds were set at a corrected *p* < 0.05, with multiple comparison correction using the AlphaSim program [56] in AFNI, determined by Monte Carlo simulation (Parameters were: single voxel p-value = 0.05, a minimum cluster size of 1.91 mm^3^, FWHM = 4 mm, within a gray matter mask corresponding to the MNI atlas).

#### Between-groups ALFF analysis

Statistical tests across groups were performed using a voxel-based, one-way analysis of covariance (ANCOVA), with age as a covariate, followed by post-hoc two-sample *t*-tests. The ANCOVA result was corrected with a cluster-level significance threshold of *p* < 0.05 (cluster size > 1.91 mm^3^ determined by a Monte Carlo simulation). The post-hoc two-sample *t*-tests were conducted within a mask showing significant differences obtained from the ANCOVA analysis, with multiple comparison correction described previously, the t-value given hereafter corresponding to the mean value of the cluster. In order to identify the effects of WMHs on the ALFF maps, a second-level analysis was performed using a two-sample t-test between SCD patients with and without silent strokes, for each cluster. All these statistical analyses were performed with AFNI functions [57].

#### Correlation analysis

To investigate the relationship between brain activity changes and neurocognitive scores, regional ALFF clusters showing significant group differences were identified for all subjects and analysis was repeated on the mean ALFF in the cluster. Pearson correlation analysis between these activities and neurocognitive scores, which were significantly lower for the SCD group than for the CTL group, were investigated. Multiple comparisons for these analyses were corrected using a false discovery rate (FDR) (*p* < 0.05) [58].

## Results

### Imaging findings

MRA was completely normal in all subjects. Abnormal WMH were documented in 1/12 ACTL patients, 4/20 SCD patients, and 0/19 CTL subjects. The number of lesions ranged from 3 to 18 and none of them were larger than 5 mm. Lesions were more common in the fronto-parietal white matter, consistent with previous descriptions [59].

Regional cerebral volumes of the CTL, ACTL and SCD groups are summarized in Table 2. All volume measurements are reported in raw values and the p-values were calculated after correcting for age and sex differences, making the group differences more apparent. After adjusting for age, sex and education level, the majority of the grey and white matter structures in the SCD group had reduced volumes (i.e., negative t-score). Several brain regions had lower white matter volume in the SCD group including the frontal (right, p = 0.02, left, p = 0.01), parietal (right, p = 0.01, left, p = 0.008) and temporal (right, p = 0.003, left, p = 0.02) lobes. No significant differences were found for grey matter volumes between the SCD and CTL groups. Cortical thickness was not different globally or regionally. Interestingly, decreases in white matter volume were nearly identical between the ACTL and SCD groups suggesting that these changes result from anemia, alone, rather than sickle cell disease-specific pathology.

**Table 2:** Cerebral volumes (expressed as mean ± standard deviation) of healthy controls (CTL), anemic control subjects (ACTL) and patients with sickle cell disease (SCD) and the p-values of SCD vs. CTL.

	CTL	ACTL	SCD	SCD vs CTL
Total Brain Volume (cm^3^)	1073 ± 246	1117 ± 249	1083 ± 256	0.01
Total Grey Matter (cm^3^)	598 ± 40	614 ± 72	620 ± 76	0.36
Total White Matter (cm^3^)	474 ± 51	503 ± 53	463 ± 52	**0.0016**
Average Thickness (mm)	4.10 ± 0.14	4.01 ± 0.16	4.43 ± 0.17	0.06
R. Frontal WM Volume	67.0 ± 8.7	73.5 ± 7.7	66.9 ± 9.0	**0.003**
L. Frontal WM Volume	65.9 ± 7.6	72.3 ± 7.6	65.1 ± 8.9	**0.0013**
R. Parietal WM Volume	37.8 ± 4.8	40.7 ± 5.0	37.0 ± 4.8	**0.0053**
L. Parietal WM Volume	44.4 ± 5.6	47.1 ± 5.9	42.6 ± 5.7	**0.0083**
R. Temporal WM Volume	37.8 ± 3.9	40.2 ± 5.5	35.7 ± 4.6	**0.0009**
L. Temporal WM Volume	31.8 ± 3.2	34.5 ± 4.3	31.8 ± 4.3	**0.014**
R. Occipital WM Volume	21.4 ± 2.7	21.2 ± 3.2	19.9 ± 3.2	0.06
L. Occipital WM Volume	22.2 ± 2.5	21.8 ± 3.1	20.3 ± 2.9	**0.04**
O_2_ Content (ml O_2_/ml blood)	13.7 ± 1.3	18.4 ± 2.0	12.7 ± 1.9	**<0.0001**
Cerebral Blood Flow (ml blood /100g/min)	85.1 ± 14.0	59.8 ± 9.4	105.4 ± 13.4	**<0.0001**
O_2_ Delivery (ml O_2_ /100g/min)	11.4 ± 1.8	10.9 ± 1.7	12.3 ± 1.7	**0.04**

Bold text indicates a statistically significant p-value. WM: white matter; ACTL: anemic controls; CTL: controls; SCD: sickle cell disease.

Phase contrast results have been summarized elsewhere [33, 34]. In our patient cohort, oxygen content was nearly 50% higher in the CTL group than in the ACTL or SCD groups. There was compensatory hyperemia in the anemic groups; cerebral blood flow was 59.8±9.4 ml/100g/min in the control subjects, 85.1±14.0 ml/100g/min in the ACTL subjects, and 105.4±13.4 ml/100g/min in the SCD subjects (p<0.0001 for CTL versus SCD or ACTL, p = 0.05 for ACTL versus SCD). Oxygen delivery (the product of cerebral blood flow and oxygen content) was slightly elevated in both patient groups, even after correction for age and sex. The difference between SCD and CTL was significant in post-hoc correction.

### Neuropsychological test performance

Statistical analysis indicated no significant differences in neuropsychological test scores between the ACTL and CTL groups. Additionally, no differences were observed between the SCD group and either the ACTL or CTL groups on the working memory, verbal memory, nonverbal memory, or social-emotional functioning measures. However, the SCD group obtained significantly lower scores, in comparison to both ACTL and CTL, on measures of nonverbal intelligence, processing speed, visuoconstructional ability, cognitive flexibility and semantic verbal fluency. Additionally, statistically significant lower scores were found for the SCD group in comparison to the CTL group on measures of verbal intelligence, phonemic verbal fluency and initial auditory attention span, as shown in Table 3.

**Table 3:** Neurocognitive scores (expressed as mean ± standard deviation) of CTL, ACTL and SCD groups and the p-values of SCD vs. CTL and SCD vs. ACTL.

	CTL	ACTL	SCD		SCD vs. CTL	SCD vs. ACTL
Full Scale IQ (FSIQ)*	98.1±17.0	97.0±8.1	90.5±10.5	**0.002**		**0.05**
Verbal Comprehension (VCI)*	98.3±15.5	96.3±10.3	93.4±9.3	**0.03**		0.25
Perceptual Reasoning (PRI)*	96.1±17.0	99.7±12.0	89.5±13.2	**0.009**		**0.03**
Block Design**	45.5±5.1	47.0±7.4	40.0±6.6	**0.03**		**0.02**
Symbol Search***	10.2±1.8	10.5±1.9	7.9±2.1	**0.02**		**0.005**
Coding***	10.0±3.6	10.5±3.8	6.9±2.3	**0.009**		**0.003**
**Trail Making Test (TMT)** ***	9.2±4.5	9.5±3.3	7.5±3.2	**0.02**		**0.02**
Verbal Fluency: Phonemic***	10.1±3.1	9.8±4.5	8.6±3.4	**0.02**		0.2
Verbal Fluency: Categories (VF-CAT)***	10.1±3.1	10.6±2.8	7.6±3.5	**0.007**		**0.012**
Verbal Fluency: Cat/SWT (VF-CAT/SWT)***	10.3±3.7	10.2±2.7	8.5±3.1	**0.02**		0.07
CVLT List A Trial 1****	-0.5±0.9	-0.8±0.9	-0.8±1.1	**0.05**		0.28

*Standard scores have a mean of 100 and a standard deviation of 15.

**T-Scores have a mean of 50 and a standard deviation of 10.

***Scaled scores have a mean of 10 and a standard deviation of 3.

**** Z-scores have a mean of 0 and a standard deviation of 1. Bold text indicates a statistically significant p-value. ACTL: anemic controls; CTL: controls; SCD: sickle cell disease.

### Whole brain functional alteration data: Differences between groups

**Fig 1:**
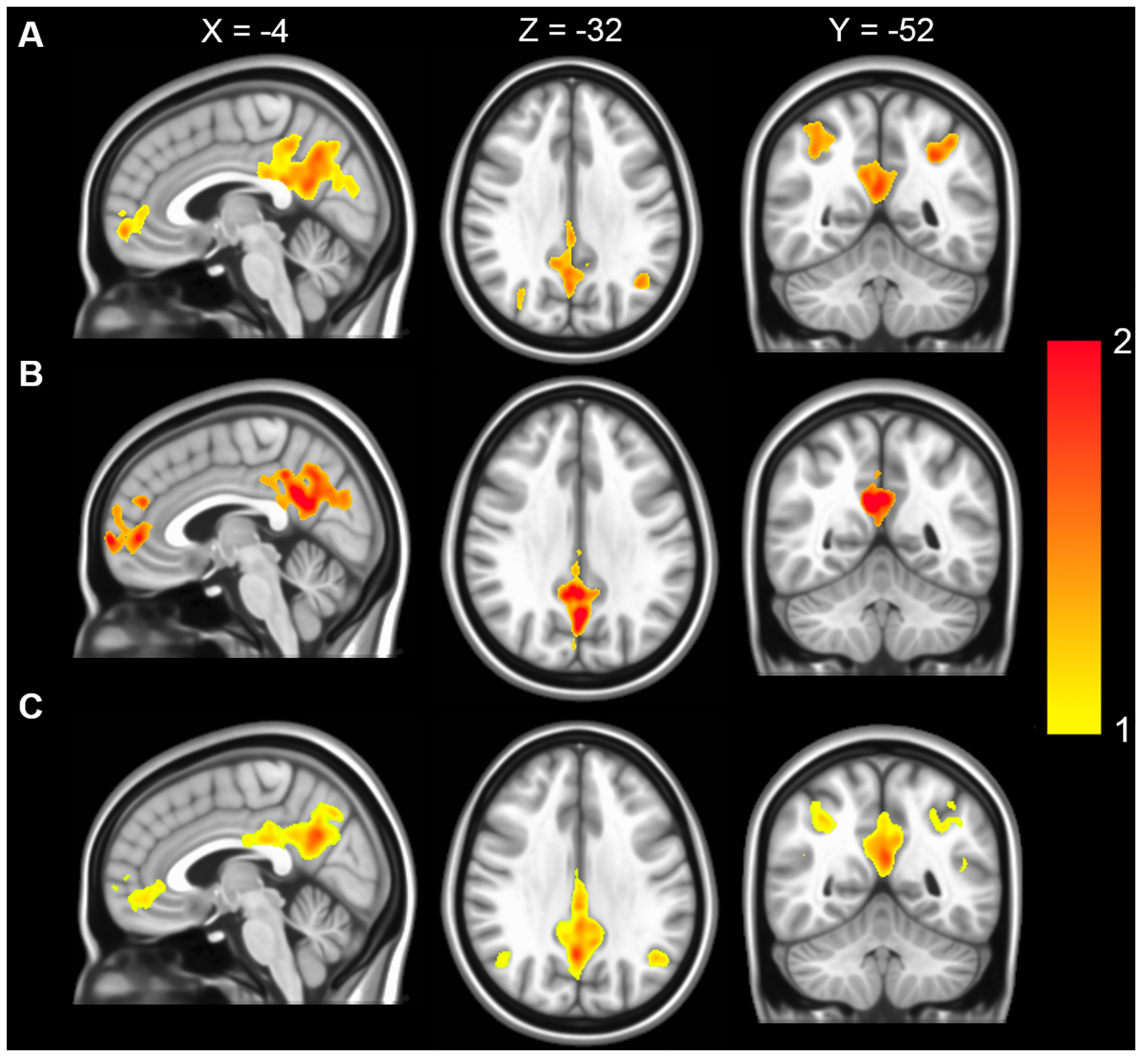
Representative one-sample *t*-test result of ALFF maps. (A) CTL subjects. (B) ACTL subjects. (C) SCD subjects. Color scale indicates t-values, resulting from one one sample t-test. Thresholds were set at a AlphaSim corrected p<0.05, determined by Monte Carlo simulation. In the Montreal Neurological Institute (MNI) template, the planes are X = -4mm, Z = -32mm and Y = -52mm for the sagittal, axial and coronal views, respectively.

**Fig 2:**
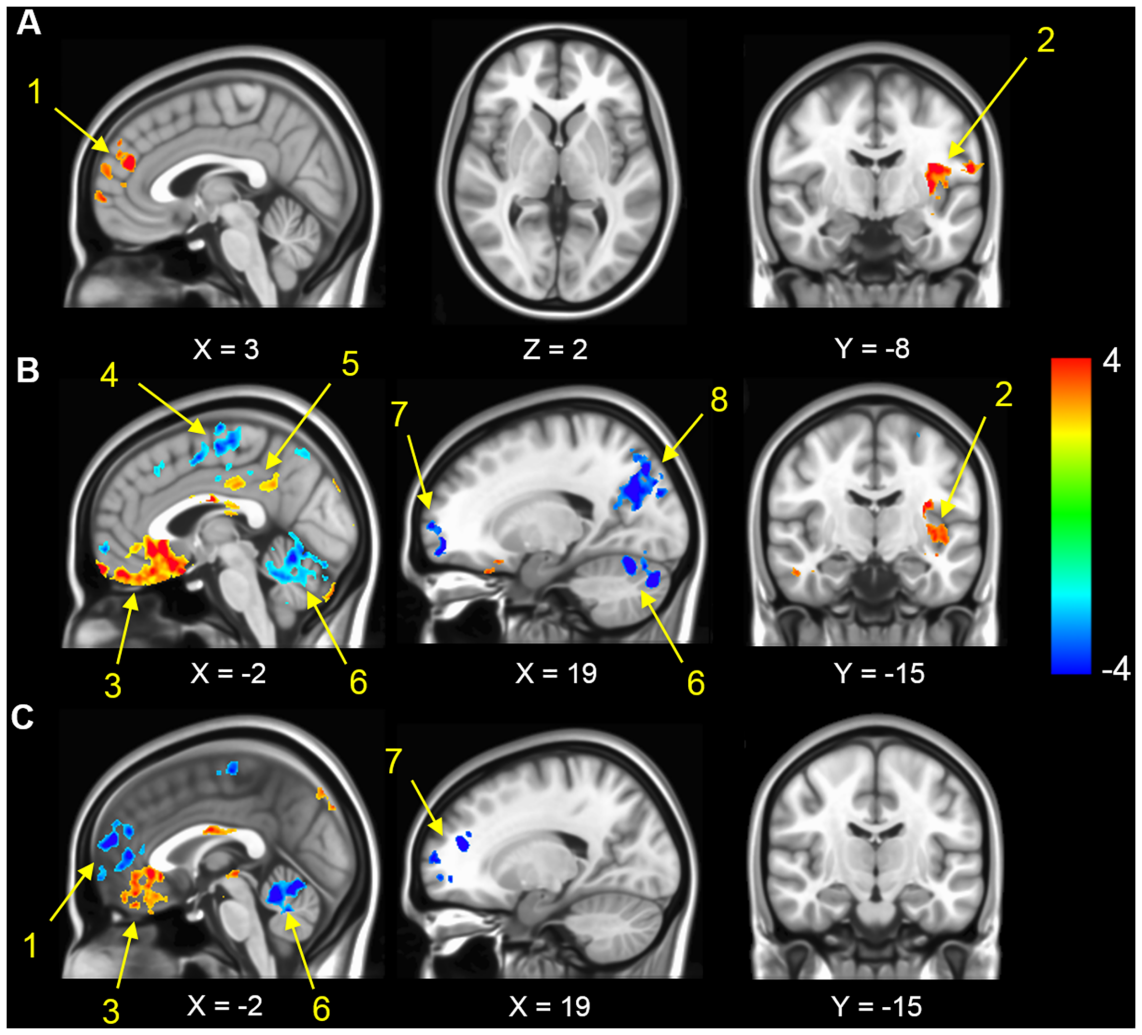
Differences in ALFF values between ACTL, SCD and CTL groups. All thresholds were set at a AlphaSim corrected p<0.05. Color scale indicates t-values, resulting from two sample t-test. Numbers and arrows represent anatomical location. Numbers and arrows represent anatomical location. A. Representative two-sample *t*-test results of ALFF maps between the ACTL versus CTL groups showing the right mSFG (arrow 1) and left insula (arrow 2). B. Representative two-sample *t*-test results of ALFF maps between the SCD versus CTL groups. Seven clusters are indicated by arrows: OFC (arrow 3), right paracentral lobule (arrow 4), PCC (arrow 5), cerebellum (arrow 6), frontal pole (arrow 7), left precuneus (arrow 8) and left insula (arrow 2). C. Representative two-sample *t*-test results of ALFF maps between the SCD versus ACTL groups. Compared with values in the ACTL group, ALFF in SCD patients increased in OFC (3) and decreased in right mSFG (arrow 1), cerebellum (arrow 6) and frontal pole (arrow 7).

No difference in activation was found between CTL groups with AA and AS phenotypes; and between the non-transfused and transfused SCD groups. As a result, AA and AS participants were combined into a single CTL group and transfused and non-transfused SCD patients were also combined together as a single SCD group. As illustrated in Fig 1, ALFF was higher than the global mean ALFF in the mPFC, PCC, precuneus, and inferior parietal lobule. Significant differences between the ACTL and CTL group were observed in the mPFC including the bilateral medial superior frontal gyri (mSFG), superior frontal gyrus and left precuneus, along with the left lingual gyrus, right postcentral gyrus, and left insula (Fig 2A). Compared with the CTL group, the SCD patients showed significantly lower activity in the left precuneus, right insula, frontal pole, superior frontal gyrus/paracentral lobule, mSFG and cerebellum. In addition, increased activity was found in the bilateral orbitofrontal cortex (OFC), bilateral anterior cingulate cortex (ACC), left insula and PCC/bilateral cingulate gyri (Fig 2B). Compared with values in the ACTL group, ALFF in SCD patients was higher in the bilateral OFC, ACC, PCC and decreased in mSFG, cerebellum, frontal pole, and right insula (Fig 2C). The detailed *t*-test results, including the size and the coordinates of the clusters are presented in Table 4.

**Table 4:** Brain areas with significant ALFF differences between the 3 groups (ACTL vs. CTL, SCD vs. CTL and SCD vs. ACTL).

Inter-group comparisons			ACTL vs. CTL		SCD vs. CTL		SCD vs. ACTL	
Cerebral regions	Right or Left	MNI coordinates	t-value	cluster size	t-value	cluster size	t-value	cluster size
**OFC**	R, L	[-4,30, -12]			5.37	37742	3.01	7748
**ACC**	R, L	[-2,30,15]			5.15	4653	2.35	2302
**Cerebellum**	R, L	[-1, -61, -30]			-2.86	15210	-5.56	9292
**Insula**	R	[38, -43,21]			-4.07	4898	-3.84	2514
	L	[-36,12, -6]	4.24	7542	4.62	14726		
**Precuneus**	L	[-9, -49,37]	-2.13	6343	-5.37	26732		
**PCC/Cingulate gyrus**	R, L	[-2, -23,34]			7.89	3070	2.58	3070
**Frontal pole**	R, L	[10,55,6]			-3.51	4490	-4.22	43200
**mSFG**	R, L	[0,26,41]	5.49	10273	-2.22	8918	-6.09	38436
**Superior frontal gyrus/Paracentral lobule**	R, L	[0, -2,51]	-3.44	1587	-2.34	7808		
**Postcentral gyrus**	R	[41, -25,51]	3.25	5002				
**Lingual gyrus**	L	[-11, -67, -7]	3.46	5002				

We reported the average t-value overall voxels in the cluster. The cluster size is given in mm^3^. Cells left blank mean no significant ALFF difference was found. Negative t-values indicate lower mean ALFF values in the first group in comparison to the second group. Positive t-values indicate higher mean ALFF values. OFC: orbital frontal cortex; ACC: anterior cingulate cortex; PCC: posterior cingulate cortex; mSFG: medial superior frontal gyrus; ACTL: anemic controls; CTL: controls; SCD: sickle cell disease.

### Effect of white matter hyperintensities

In our study, WMHs were observed in 4/20 SCD patients in the 3D T2-weighted images. As described in the methods section, a comparison analysis restricted to clusters with significant activation differences was performed between SCD patients with and without WMHs, to assess the relationship of this brain damage on functional activity. As reported in Table 5, decreased activity was observed in small areas in precuneus, frontal pole, mSFG and cerebellum. The SCD patients with silent strokes showed significantly higher activity in a small cluster in the right insula.

**Table 5:** Statistic results between the SCD patients with and without WMHs.

Inter-group comparisons			SCD with WMHs vs.	
			SCD without WMHs	
Cerebral regions	Right or Left	MNI coordinates	t-value	cluster size
**OFC**	R, L	[-4,30, -12]		
**ACC**	R, L	[-2,30,15]		
**Cerebellum**	R, L	[-1, -61, -30]	-2.61	839
**Insula**	R	[38, -43,21]	3.21	1599
	L	[-36,12, -6]		
**Precuneus**	L	[-9, -49,37]	-2.98	2918
**PCC/Cingulate gyrus**	R, L	[-2, -23,34]	NS	
**Frontal pole**	R, L	[10,55,6]	-2.61	965
**mSFG**	R, L	[0,26,41]	-3.09	550
**Superior frontal gyrus/Paracentral lobule**	R, L	[0, -2,51]		

The t-value corresponds to the mean value of the cluster. The cluster size is given in mm^3^. Cells left blank mean no significant ALFF difference was found. OFC: orbital frontal cortex; ACC: anterior cingulate cortex; PCC: posterior cingulate cortex; mSFG: medial superior frontal gyrus; ACTL: anemic controls; CTL: controls; SCD: sickle cell disease.

### Correlation of functional activity and neurocognitive scores

As explained in the method section, the relationship between abnormal ALFF and significantly lower neurocognitive performance in the SCD group was investigated by Pearson correlation analysis (Table 6). Associations between ALFF in some cerebral regions and neurocognitive scores, such as VCI, FSIQ, VF-CAT/SWT and TMT, were detected; however, after false discovery rate (FDR) correction, significant group differences were found only for the frontal lobe and VF-CAT/SWT.

**Table 6:** Correlation results between regional mean ALFF and neurocognitive scores.

Cerebral regions	Neurocognitive test	Pearson’s correlation	p-value (FDR correction)
**ACC**	**VCI**	0.52	0.025
	**FSIQ**	0.52	0.025
	**VF-CAT/SWT**	0.51	0.029
**Insula**	**TMT**	0.47	0.044
	**VCI**	-0.22	0.044
**Frontal pole**	**VF-CAT/SWT**	-0.0015	**0.0028(0.028)**
	**FSIQ**	-0.14	0.028

Only one correlation between frontal pole and VF-CAT/SWT survived FDR correction. ACC: anterior cingulate cortex; VCI: verbal comprehension; FSIQ: full-scale intelligence quotient; VF-CAT: verbal fluency: catergories; SWT: Stroop word test; TMT: trail-making test.

## Discussion

### fMRI findings

To our knowledge, this is the only study to date investigating the effect of SCD on whole brain spontaneous activity using amplitude of low frequency fluctuation (ALFF) analysis. The default mode network was identified in similar regions across the SCD, CTL, and ACTL groups in this study, and consistent with previous observations in healthy control subjects [21]. Indeed, we observed that a higher activity, detected by the ALFF method, was found in the PCC, precuneus, mPFC and the angular parietal cortex, for the three groups. Nonetheless, we observed many significant functional differences between the SCD and CTL groups. ALFF differences were symmetric in most of the brain areas, but we observed lateralization in the precuneus and insula.

**Fig 3:**
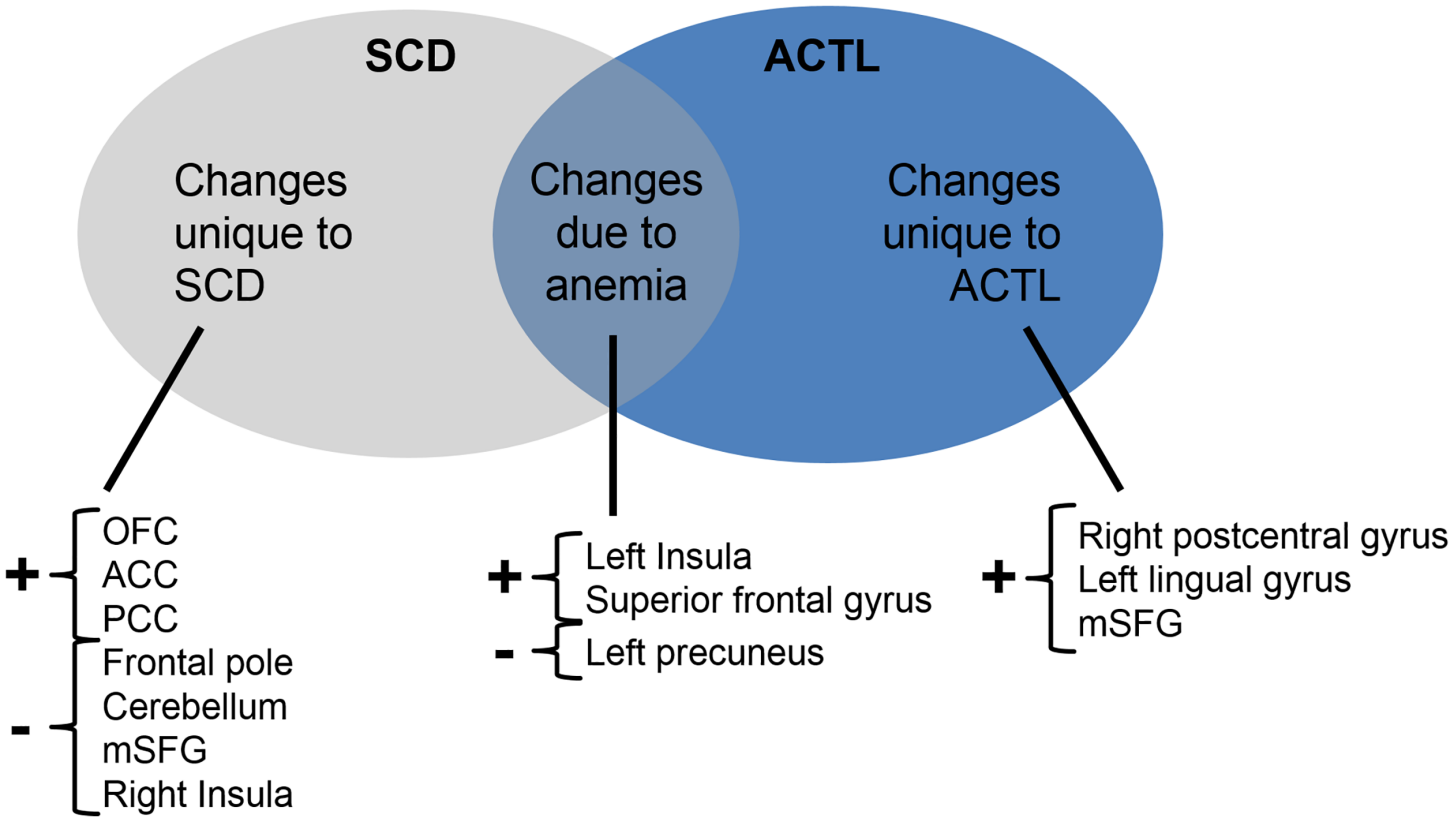
Venn diagram representing changes (from normal) seen in SCD and ACTL groups. The changes are grouped with regards to their cause. They could be either unique to SCD patients (left/grey), common to SCD and ACTL patients (middle) or unique to ACTL patients (right/blue). OFC: orbital frontal cortex; ACC: anterior cingulate cortex; PCC: posterior cingulate cortex; mSFG: medial superior frontal gyrus.

Sickle cell disease is strongly associated with low hematocrit, increased cerebral blood flow (CBF) and increased cerebral blood volume (CBV) which could impact the BOLD signal in SCD [59, 60]. It was therefore essential to separate the ALFF changes that are only due to anemia (or its physiological consequences) from other SCD-specific abnormalities. Fig 3 classifies the ALFF changes into those unique to SCD or ACTL and those common to both groups. The ALFF changes common to the two groups, located in the left insula, left precuneus and superior frontal gyrus, would explain functional changes to the brain caused by anemia alone. Regions, such as the superior frontal gyrus and precuneus, are within brain regions as known as watershed zones, because they are located between the vascular territories of major cerebral arteries [6]. These watershed zones are vulnerable to acute drops in oxygen delivery (e.g. from sleep apnea, acute anemia) especially in light of diminished autoregulatory cerebrovascular flow reserve due to basal hyperemia [33, 34]. Repeated ischemia-reperfusion injury may be responsible for decreased activity in watershed cortical areas [61]. Interestingly, we observed comparable loss of white matter in the ACTL group and SCD patients in the anterior and middle cerebral artery territories, consistent with chronic intermittent hypoxia in these regions.

On the other hand, unique differences in ALFF seen in the ACTL group but not SCD group could be indicative of compensatory mechanisms which might be impaired by the additional complications beyond chronic anemia found in SCD. The right postcentral gyrus and left lingual gyrus was uniquely increased in ACTL Similarly, ALFF in mSFG was significantly increased in ACTL, relative to control, but significantly decreased in SCD subjects. We postulated that the regions where ALFF were higher only in ACTL subjects reflect primary responses to anemia where SCD patients would have had disease-specific changes that blunt ALFF increases within these regions.

ALFF was also uniquely lower in SCD patients in the frontal pole, cerebellum, mSFG and right insula, consistent with disease-specific ALFF inhibition. The core of infarcted cortical regions due to chronic ischemia and stroke generally shows decreased relative ALFF [28, 62]. However, none of our subjects had known prior stroke or evidence of cortical strokes on T1 or T2 imaging. Furthermore, we did not identify any areas of cortical thinning between SCD and CTL subjects in regions with abnormal ALFF, as reported in [12, 63]. Although ALFF reflects cortical activity, smaller white matter damage, such as silent stroke could also potentially decrease ALFF by deafferentation [64, 65]. Small white matter hyperintensities that lack overt neurological symptoms, are common in SCD patients [9, 10, 66, 67] and were observed in 4/20 of SCD patients. Historically, the most common areas for silent strokes are the frontal (78%), parietal (51%) and temporal lobes (15%) [68] and we observed a similar distribution. Additionally, lower gyral white matter in watershed regions was previously reported in a study of SCD patients which may be indicative of preclinical white matter injury [12]. In [61], control temporal-concatenation group ICA analysis was not able to detect connectivity differences among SCD patients according to WMH volume. Contrary to this result, decreased ALFF was observed in small areas in the frontal pole and mSFG between patients with and without silent stroke. Silent strokes are a common manifestation of more generalized white matter diseases in SCD patients, so it is not surprising that their primary location was associated with reduced ALFF.

In contrast, increased ALFF (unique to SCD) was seen in the ACC, PCC, and OFC. Previous fMRI studies in patients with chronic pain have described the ACC as a central player in the pain network, whose activation is associated with the emotional response to pain [69]. Ma et al. exhibited a higher activation in the cingulate gyrus, including the ACC, in SCD subjects [70] and attribute these changes to SCD patients’ experiences with pain crises. Thus one possibility is that the increased ALFF observed in the ACC in our study may contributed to the increased the chronic pain experienced by SCD patients [71].

However, half of our patient cohort was chronically transfused for stroke prophylaxis and their pain exposure is minimal compared with the non-transfused patients. Furthermore, no resting state differences were observed between transfused and nontransfused subjects, decreasing the likelihood that prior pain history was a key driver in the ALFF differences.

An alternative hypothesis is that the ACC, insula and frontal pole have been implicated as cortical regulators of autonomic nervous system function [62]. Abnormal activity in these areas could potentially explain the dysautonomia, previously described in SCD patients [64, 65, 72, 73]. Studies have reported altered sympathetic-vagal balance (i.e. decreased parasympathetic activity and increased sympathetic activity) [74, 75]. More specifically, while the dorsal insula was bilaterally associated with parasympathetic regulation, the right ventral insula showed sympathetic predominance. The sympatho-vagal balance may be affected by the opposite abnormal activity found in the right and left insula. In the future, the use of validated pain surveys and assessments of autonomic function might help decipher these two possibilities.

### Neuropsychological assessment

SCD patients attained lower scores on measures of verbal and nonverbal reasoning, processing speed and executive functions. However, while statistically significant, most comparisons yielded an approximately one half to two-thirds standard deviation difference in performance between the SCD group and the control groups, suggesting that these differences are subtle. Our observations are in accordance with prior research noting a similar level of discrepancy on neurocognitive measures between patients with SCD without a known history of overt or silent strokes and controls [76–78]. For example, consistent with our findings, in a review of the literature, Kawadler et al. reported that groups with SCD and no history of stroke have a mean difference of 7 IQ points compared to healthy control groups [79].

Higher resting state activity in the frontal lobe was associated with lower performance on verbal fluency tasks involving semantic retrieval and cognitive flexibility. This is consistent with the role of the mPFC in executive control [80].

### Potential vascular confounders on the BOLD signal

Although fMRI has been used extensively in the investigation of human brain function, it fundamentally transduces vascular activity, and most mathematical models of BOLD response are modulated by hematocrit levels and resting cerebral blood flow. SCD disease causes abnormal cerebrovascular changes including increased CBF, CBV, microvascular vasomotion and obstruction, all of which could potentially influence ALFF independent of neural activity. Previous works using breath-holding or CO_2_ inhalation in non-SCD subjects have demonstrated increased CBF to the levels seen in SCD subjects [81], producing a 20% reduction in spontaneous BOLD magnitude [82–84]. The mechanism is postulated to be a loss of cerebrovascular vasodilatory reserve (CVR). CVR is known to be blunted in SCD [35, 67] and BOLD responses to visual stimulation are also diminished [85]. In the present study, we did not observe systematically decreased ALFF in anemia patients except in the precuneus and superior frontal gyrus. In contrast, previous studies [86–88] suggest that decreased hematocrit, even among non-anemic subjects, may cause regional increases in the mPFC, ACC and intraparietal sulcus regions, as well as in the insular and opercular cortex [89]. Our findings are in agreement with previous studies in terms of the ACC and insula, but contradictory in the mPFC.

Globally, changes in CBV overlap with the changes in CBF. CBV could have a larger impact on BOLD response in SCD patients because the fMRI signal depends on the total amount of deoxyhemoglobin within each small volume of tissue. In fact, the venular density has a significant impact on the observed amplitude of the BOLD signals [28, 90], with higher spontaneous activity observed in the vein-dense regions such as the cuneus, precuneus, culmen and frontal gyri, than other grey matter regions [91]. In our study, we observed increased fluctuations in culmen for the SCD group. However, the opposite results, predicted based on CBV arguments, were found in the precuneus and superior frontal gyrus for ACTL and SCD groups.

Lastly, we cannot exclude the role that spontaneous microvascular oscillations, increased oxygen extraction, or microvascular occlusion have in increasing ALFF in SCD patients, independent of neural activity. Myogenic oscillations (0.8Hz) are characteristically increased in SCD patients and were discovered by analyzing the cutaneous blood flow in the forearm. This periodic flow may be present to compensate for the deleterious altered rheology of erythrocytes containing polymerized hemoglobin S [92, 93]. In SCD, the reduced deformation of blood cells increases the apparent viscosity of blood flow through the microcirculation, potentially increasing oxygen extraction and BOLD signaling. Both abnormal blood rheology and microvascular occlusion could contribute to regional differences in ALFF, independent of neuronal activity.

### Limitations

As summarized above, SCD causes abnormal cerebrovascular changes which could potentially influence ALFF, independent of neural activity. Regional measurements of CBF, CBV, and venular density may help further remove the vascular contribution to BOLD signal fluctuations.

While ALFF changes common to ACTL and SCD likely represent a consequence of chronic anemia, we cannot discern between normal physiologic compensation (such as increased cerebral blood flow or volume) or pathological changes (such as microvascular damage). However, the equal white matter loss observed in ACTL and SCD patients suggests that chronic anemia, by itself, is harmful to the brain.

The link between white matter hyperintensities and ALFF changes was underpowered and should be interpreted as hypothesis generating rather than conclusive. Our SCD group was also heterogeneous, potentially increasing variability. However, the patient heterogeneity is also a strength of this study because it disrupts coincidental associations. For example, the transfused and non-transfused SCD patients had identical ALFF changes in brain regions previously attributed to chronic pain, despite very different pain exposures in the two groups, suggesting that pain is unlikely to be primary etiology.

Additionally, it is difficult to make direct causal inferences regarding the relationships between ALFF and neurocognitive scores. Indeed, most of these correlation results were not significant after the multiple comparison correction. Due to the limitation of our dataset (only 12 ACTL subjects), these two groups were not matched in terms of maternal education.

Lastly, we are only reporting group differences and the prognostic value for any individual remains uncertain. Thus it is premature for us to conclude that ALFF changes are useful for patient management.

## Conclusions

We investigated the effect of SCD on spontaneous brain activity using an ALFF analysis. This study was strengthened by controlling for anemia using a separate patient cohort (ACTL). ALFF was not different in sickle cell trait and hemoglobin AA controls. It also did not differ between SCD patients with or without chronic transfusion therapy. The resting state activity changes located in left insula, mSFG and left precuneus, were common anemic subjects, with or without,sickle cell hemoglobin. Patients with sickle cell disease, however, demonstrated increased activity in the OFC, ACC and PCC, and decreased activity in the frontal pole, cerebellum and mSFG that persisted after controlling for anemia. These abnormal ALFF patterns occurred within the pain network, cortical regulators of autonomic nervous system function, as well as regions affected by structural damage such as strokes. Abnormalities in the frontal pole activities were correlated with VCI and VF-CAT, leading to the hypothesis that ALFF differences in these areas represent a biomarker of SCD-related cognitive dysfunction. These findings may lead to a better understanding of the pathophysiology of SCD.
